# TaxSEA: rapid interpretation of microbiome alterations using taxon set enrichment analysis and public databases

**DOI:** 10.1093/bib/bbaf173

**Published:** 2025-04-21

**Authors:** Cong M Pham, Timothy J Rankin, Timothy P Stinear, Calum J Walsh, Feargal J Ryan

**Affiliations:** Flinders Health and Medical Research Institute, University Drive, Flinders University, Bedford Park, SA 5042, Australia; Flinders Health and Medical Research Institute, University Drive, Flinders University, Bedford Park, SA 5042, Australia; Department of Microbiology and Immunology, Doherty Institute, University of Melbourne, 792 Elizabeth St, Melbourne, VIC 3000, Australia; Centre for Pathogen Genomics, Doherty Institute, University of Melbourne, 792 Elizabeth St, Melbourne, VIC 3000, Australia; Department of Microbiology and Immunology, Doherty Institute, University of Melbourne, 792 Elizabeth St, Melbourne, VIC 3000, Australia; Centre for Pathogen Genomics, Doherty Institute, University of Melbourne, 792 Elizabeth St, Melbourne, VIC 3000, Australia; Flinders Health and Medical Research Institute, University Drive, Flinders University, Bedford Park, SA 5042, Australia; Precision Medicine, South Australian Health and Medical Research Institute (SAHMRI), North Terrace, Adelaide, SA 5000, Australia

**Keywords:** metagenomics, microbiota, microbiome, differential abundance, microorganisms

## Abstract

Microbial communities are essential regulators of ecosystem function, with their composition commonly assessed through DNA sequencing. Most current tools focus on detecting changes among individual taxa (e.g. species or genera), however in other omics fields, such as transcriptomics, enrichment analyses like gene set enrichment analysis are commonly used to uncover patterns not seen with individual features. Here, we introduce TaxSEA, a taxon set enrichment analysis tool available as an R package, a web portal (https://shiny.taxsea.app), and a Python package. TaxSEA integrates taxon sets from five public microbiota databases (BugSigDB, MiMeDB, GutMGene, mBodyMap, and GMRepoV2) while also allowing users to incorporate custom sets such as taxonomic groupings. In silico assessments show TaxSEA is accurate across a range of set sizes. When applied to differential abundance analysis output from inflammatory bowel disease and type 2 diabetes metagenomic data, TaxSEA can rapidly identify changes in functional groups corresponding to known associations. We also show that TaxSEA is robust to the choice of differential abundance analysis package. In summary, TaxSEA enables researchers to efficiently contextualize their findings within the broader microbiome literature, facilitating rapid interpretation, and advancing understanding of microbiome–host and environmental interactions.

## Graphical Abstract

**Graphical Abstract:**
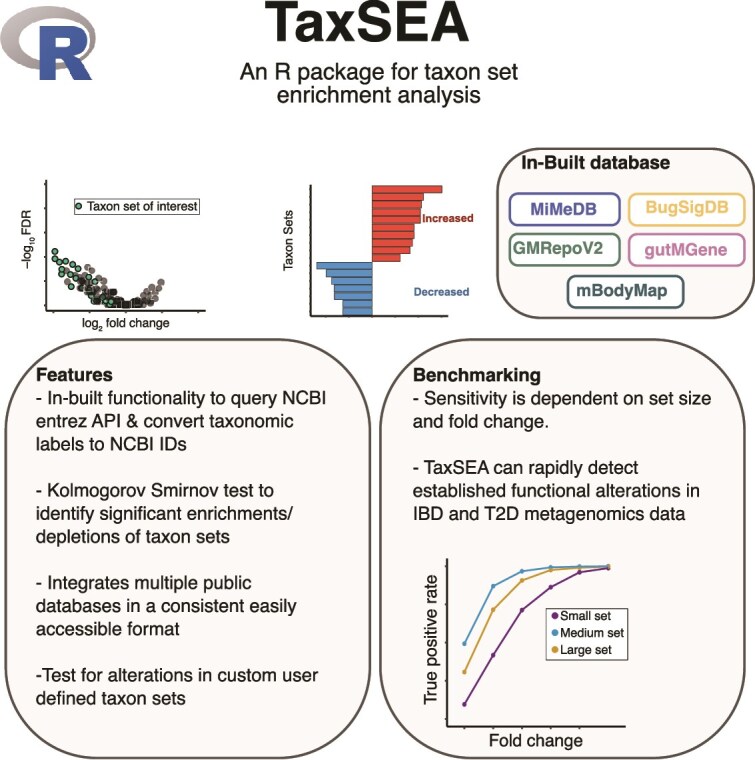

## Introduction

Host-associated microbial communities, particularly the gut microbiota, play pivotal roles in immune regulation [1], drug metabolism [2], neural development [3], and nutrition [4] and are therefore frequently studied in medical research. The assessment of the gut microbiota commonly involves DNA sequencing, often with the aim of comparing the microbial composition between samples. This comparison is achieved through differential abundance (DA) analysis, which identifies taxa (e.g. species, genera, operational taxonomic units) which are present at varying levels between groups of samples (e.g. treatment vs. control). However, interpreting alterations identified through DA analysis in humans is complex.

**Figure 1:**
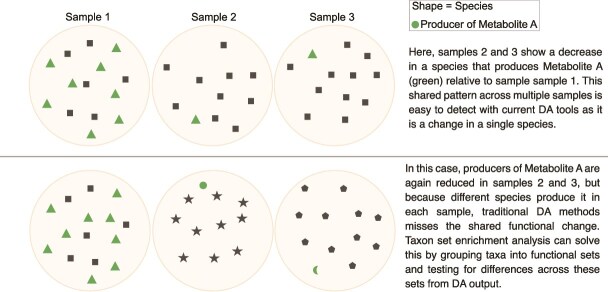
Example of the utility of taxon set enrichment analysis for detecting shared functional changes missed by traditional differential abundance methods.

Microbial taxa are not independent entities, yet standard DA analysis treats them as isolated units, overlooking the complex phylogenetic and functional relationships that shape microbial communities. While closely related species often share metabolic functions, they may also exhibit important functional differences. When DA analysis aggregates read counts at higher taxonomic levels (e.g. genus or family), opposing shifts in individual species may cancel each other out, obscuring meaningful biological patterns. For example, antibiotic treatment may suppress certain species while allowing resistant species within the same genus to expand and occupy the vacant niche, creating a significant ecological shift that appears as no net change at the genus or family level. This is illustrated with antibiotic resistance profiles that can vary considerably between species in the genus *Enterococcus* [5]. Conversely, functional redundancy allows distantly related microbes to perform the same role, meaning that taxonomic shifts may seem disconnected even when they result in the same functional outcome [6]. For instance, a reduction in one butyrate-producing species may be offset by an increase in another, making it difficult to detect functional changes through taxonomic analysis alone (Fig. 1). To address this, functional profiling tools such as gutSMASH [7] and HUMAnN3 [8], profile gene clusters and pathways from metagenomic data and construct additional count matrices for further analysis. While powerful, these approaches suffer from sparsity and high dimensionality issues in addition to requiring significant compute resources and analysis time.

In the past, DA analysis was performed using either simple statistical tests (e.g. Wilcoxon rank sum test), or by adapting differential gene expression approaches from transcriptomics (e.g. edgeR, DESeq2 [9]) although there are now multiple dedicated DA tools such as ALDEx2 [10], fastANCOM [11], and LinDA [12]. Results from DGE analyses are routinely further explored using gene set enrichment analyses (GSEAs), which identify changes in sets of genes representing pathways (e.g. cell cycle, oxidative phosphorylation), disease associations, or other functional groupings (e.g. genes regulated by the same transcription factor). This process was originally based upon the non-parametric Kolmogorov–Smirnov (KS) test which assesses whether the distribution of members in a predefined gene set differs from the overall dataset distribution [13]. GSEA takes seconds to run, requires minimal compute resources, and allows for rapidly contextualizing findings and generating hypotheses from DGE analysis output. Taxon set enrichment analysis (TSEA) applies this concept to microbial communities by grouping taxa into predefined functional or taxonomic sets. Instead of examining individual taxa in isolation, TSEA can test whether these groups, or ‘taxon sets,’ show significant changes.

We could identify two implementations of TSEA-type methods in the literature: the TSEA module in the MicrobiomeAnalyst pipeline [14, 15] and the microbe-set enrichment analysis (MSEA) tool [16]. MSEA calls upon a combination of taxon sets defined by literature mining together with the Disbiome database [17], whereas TSEA incorporate sets from a variety of sources [14, 15]. There is also competitive balances for taxonomic enrichment analysis (CBEA) [18], which offers the ability to generate enrichment scores for taxon sets in individual samples. While useful, current tools have some limitations. They do not allow users to easily incorporate custom sets or additional public databases, and those that perform statistical testing rely on an over-representation analysis (ORA) framework, which tests for overlap between input taxa and predefined sets using statistical methods such as Fisher’s exact test, hypergeometric test, or Chi-squared test. A major drawback of ORA is that it requires strict inclusion thresholds (e.g. *P*-value or fold change), which may exclude taxa whose DA are not significant on their own but collectively contribute to enrichment patterns. In RNA-Seq analysis, both ORA and GSEA are commonly used, as they serve different purposes. However, no equivalent GSEA-like approach has been widely adopted for microbiome research, which provide a more flexible enrichment analysis framework.

To address this gap, we have developed TaxSEA, a framework for TSEA that directly utilizes output from common DA analysis tools. TaxSEA is built on a GSEA-like framework, enabling the exploration of changes in user-defined taxon sets or predefined sets from five public microbiota databases, covering disease signatures, metabolite producers, and previously published associations. TaxSEA can query the NCBI Entrez Application Programming Interface (API) to convert between taxonomic naming schemas and is robust to the choice of DA analysis software. TaxSEA is available as packages in R and Python and a web portal at https://shiny.taxsea.app.

## Materials and methods

TaxSEA is an open-source framework under the GNU GPL-3 licence. TaxSEA takes as input species or genus names together with a rank/value for each taxon (e.g. log2 fold change, correlation coefficient, etc.). TaxSEA then utilizes the non-parametric KS test to assess whether the distribution of members in a predefined set differs from the overall distribution. Prior to testing TaxSEA filters input sets to only include those with a minimum number of taxa detectable in the input data (user settable, default = 5). A *P*-value is computed with the KS test for each taxon set and adjusted using the Benjamini–Hochberg procedure to control the false discovery rate (FDR). The output of TaxSEA is a table containing the name of the taxon set, the median rank for the detected members, the *P*-value, FDR, and members of that set which are detected in the data. This approach is adapted from the original GSEA from transcriptomics [13] While TaxSEA can used with any input sets the user provides, for convenience and ease of use we have provided a database (TaxSEA-DB) constructed from the following sources:

- BugSigDB: a community-editable database of manually curated microbial signatures from published DA studies [19]
- GMrepo v2: a curated human gut microbiome database with special focus on disease markers and cross-dataset comparison [20]
- gutMGene: a comprehensive database for target genes of gut microbes and microbial metabolites [21]
- mBodyMap: a curated database for microbes across human body and their associations with health and diseases [22]
- MiMeDB: the Human Microbial Metabolome Database [23]

For each source above, taxon sets were downloaded, taxonomic identifiers converted to NCBI taxonomic IDs by querying the NCBI Entrez API, made non-redundant and transformed into a named list of taxon sets in R where each element is a named set and the members NCBI taxonomic IDs. As MiMeDB does not provide API access or bulk downloads, a manually curated subset of taxon sets was downloaded. Similarly, only a subset of body sites from mBodyMap were included (oral cavity, skin, and vaginal tract) [24].

To assess TaxSEAs sensitivity, we used a signal implantation approach, a method shown to be effective for benchmarking DA methods [25]. This approach involved introducing known enrichment signals into real metagenomic fold change data and evaluating TaxSEA’s ability to recover these signals. We first generated a set of null distributions by selecting random, non-overlapping subsets of healthy adult samples (*n* = 25 each) from the LifeLines-DEEP cohort (LLD; *n* = 1040), obtained via the curatedMetagenomicData R package (v3.1) [26]. Fold changes were generated through DA analysis performed on each subset using LinDA (MicrobiomeStat R package v1.1) [12]. This process was repeated 1000 times, creating a collection of distributions representing expected random variability in microbiome datasets. Any distributions exhibiting pre-existing taxon set enrichments (FDR < 0.05) were excluded to ensure that our null distributions contained no systematic enrichment. Next, we systematically implanted signals across all taxon sets that met our defined size criteria: small (<10 taxa), medium (10–50 taxa), and large (>50 taxa). Rather than selecting a subset of taxon sets, we implanted signals into all available sets within these size categories to provide a broad and unbiased evaluation of TaxSEA’s sensitivity. For each set, a randomly selected null distribution was modified by adjusting the log2 fold changes of taxa within the chosen set to create an enrichment signal using the runif() function in R, which draws values from a uniform distribution within predefined minimum and maximum bounds based on the tested effect size (1.5× fold change; 2× fold change; 2.5× fold change; 3×, 4×, and 5×). This procedure was repeated 1000 times.

To evaluate TaxSEA’s ability to detect biologically relevant associations, we applied TaxSEA to publicly available metagenomic datasets obtained from the curatedMetagenomicData R package (v3.1) [20]. Two datasets were selected for analysis: Human Microbiome Project [27] and the faecal metagenome of type 2 diabetes (T2D) patients [28]. DA analysis was performed using LinDA with both datasets. For the comparison between DA packages, fastANCOM (v0.0.4) and ALDEx2 (v1.34.0) both on default parameters were used. MSEA (v0.0.2) was performed in Python (v3.11.8). TSEA was run in the MicrobiomeAnalyst webtool using the microbiome-metabolite taxon sets (http://microbiomeanalyst.ca/MicrobiomeAnalyst). See ‘Data Availability’ section for code and underlying data. Benchmarking, code for creating figures and simulations can also be found on GitHub (https://github.com/feargalr/TaxSEA_benchmarking).

## Results and discussion

**Figure 2:**
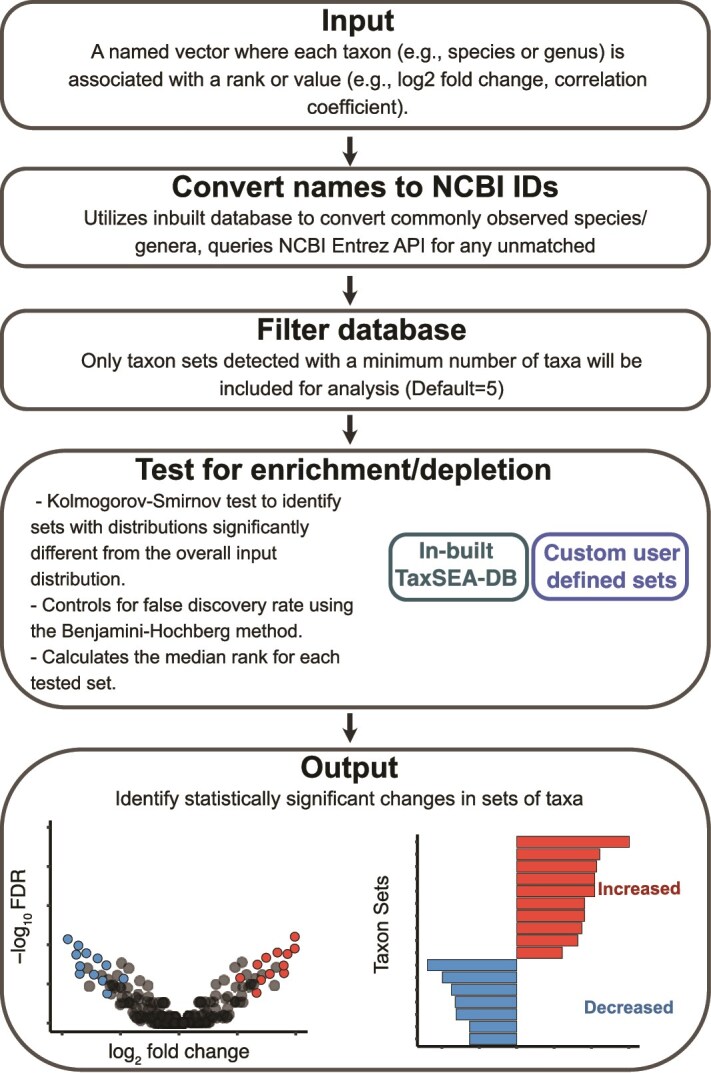
Overview of the taxon set enrichment analysis workflow using TaxSEA.

TaxSEA is an analysis package available in R, Python and through a web portal to identify enrichments or depletions of sets of taxa (Fig. 2). For ease of use, TaxSEA includes a database (TaxSEA-DB; Fig. 2a), which is collated from multiple public sources. In total this database contains 2529 taxon sets made up of 2785 individual taxa from GMRepoV2 [20], GutMGene [21], mBodyMap [22], MiMeDB [23], and BugSigDB [19]. During the development of TaxSEA, we found that public databases disproportionately favour the human gut microbiota. Therefore, our focus here is on evaluating TaxSEA to interpret DA analysis in that context. Nonetheless, TaxSEA can in principle handle samples from any environment and offers users the flexibility to add custom taxon sets for testing through its custom-db feature. To aid with interpretation and usability, we have broadly divided taxon sets from these databases into three categories for reporting enrichment signatures—health and disease signatures (GMRepoV2, mBodyMap), metabolite producers (MiMeDB, GutMGene), and previously published microbiota associations (BugSigDB). Select taxon sets from mBodyMap covering non-gut sites (mouth, skin, vaginal tract) were included as the presence of non-gut associated bacteria can be a feature of dysbiosis [29, 30].

**Figure 3:**
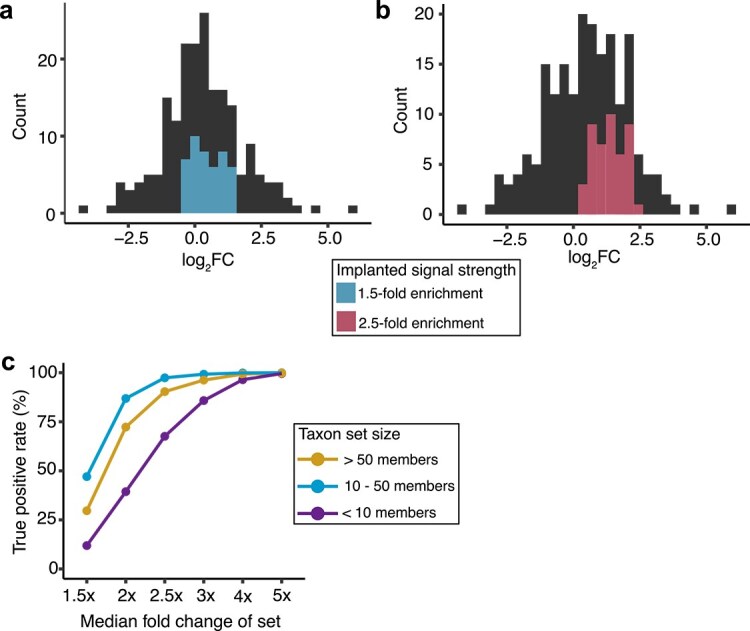
Representative examples from in silico signal implantation assessments, displaying fold change distributions with (a) a 1.5× increase and (b) a 2.5× increase for a taxon set. (c) TPR of TaxSEA at different fold changes and taxon set sizes.

We first sought to evaluate whether TaxSEA is accurate. While many metagenomic analysis tools have been benchmarked with efforts like the Critical Assessment of Metagenome Interpretation [31], enrichment analysis presents additional challenges. For example, a single bacterial species will produce many metabolites, often as part of related metabolic processes, and certain species develop cross-feeding relationships and so may form a co-occurring group. Developing a ground truth dataset in this context is challenging as such overlaps represent biological realities but complicate the calculation of false negatives from enrichment testing. As such we assessed the accuracy of TaxSEA by first evaluating sensitivity (i.e. true positive rate, TPR) with an in silico approach, and second by testing the ability of TaxSEA to recover known and biologically plausible signals in real data. TPR was evaluated by comparing randomly selected subsets from the LLD (*n* = 1040) and implanting an enrichment signal. For example implanting a set with a median fold-change of 1.5× (Fig. 3a) or 2.5× (Fig. 3b). The TPR of TaxSEA improves with higher fold changes and larger set sizes, reflecting the algorithm’s ability to capture more prominent biological signals effectively (Fig. 3c). For taxon sets with fewer than 10 members, the TPR was 11.9% at a 1.5× fold change but increased to 85.8% at 3× fold change. In medium-sized taxon sets (10–50 members) the performance improves substantially, with a TPR of 47.01% at 1.5×, 86.9% at 2×, and >97% beyond 2.5× (Fig. 3c). Interestingly, larger taxon sets (more than 50 members) show a poorer performance than medium sets with TPR of 29.7% at a 1.5× change and 72.3% at 2×, potentially reflecting a dilution of signal as the size of the set nears the size of the background, making it more difficult to differentiate between the two distributions. Overall, this signal implantation evaluation found that TaxSEA demonstrates high sensitivity when taxon sets have a greater than 2.5× change, particularly in sets larger than 10 members.

**Figure 4:**
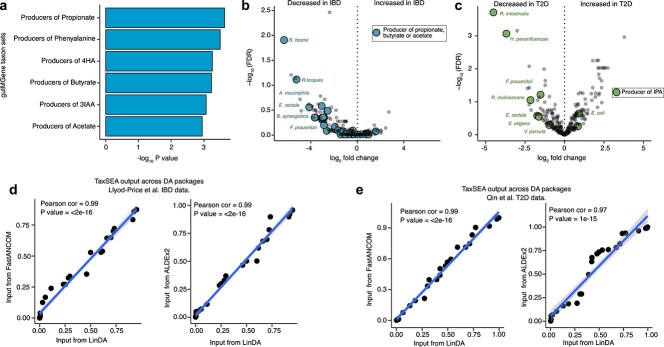
(a) Bar plot of significantly depleted taxon sets (FDR < 0.1) in IBD compared to controls in the Lloyd-Price et al. dataset. Abbreviations: 4HA = 4-hydroxyphenylacetic acid; 3IAA = indole-3-acetic acid. (b) Volcano plot showing depletion of SCFA producers (i.e. propionate, butyrate, or acetate producer taxon sets) from the Lloyd-Price et al. dataset. (c) Volcano plot highlighting the depletion of producers of IPA in T2D patients from the Qin et al. dataset. (d) Scatter plots showing correlations between *P*-values from TaxSEA across three differential abundance analysis tools (LinDA, FastANCOM, and ALDEx2) using (d) Lloyd-Price et al. dataset and (e) Qin et al. dataset.

Next, we sought to evaluate whether TaxSEA could detect biologically plausible associations in real data from the curatedMetagenomicData R package [26]. To avoid issues with overlapping testing and training data, we excluded disease signatures from this evaluation as GMRepoV2 is built from public metagenomic datasets. First, we re-analysed data from the second phase of the Human Microbiome Project [27] that compared the faecal metagenome of inflammatory bowel disease (IBD) patients (*n* = 99) and controls (*n* = 27). DA analysis with LinDA identified five species which were significantly decreased among IBD patients compared to controls (FDR < 0.1; Supplementary Table S1, *Roseburia hominis, Ruminococcus torques, Ruminococcus bromii, Firmicutes bacterium CAG 83*, and *Roseburia* sp. *CAG 18*2). TaxSEA detected significant (FDR < 0.1, median absolute fold change >2.5×) alterations in 6 taxon sets marked by a depletion in producers of short-chain fatty acids (SCFA) such as butyrate, acetate, and propionate driven by decreases in taxa such as *Faecalibacterium prausnitzii*, multiple *Roseburia* spp., *Prevotella copri*, among others (Supplementary Table S2 and Fig. 4a). A depletion of SCFA producers is hallmark of the IBD microbiota [32]. Notably, several of these SCFA-producing species alone did not meet the significance threshold (FDR < 0.1) (Supplementary Table S1 and Fig. 4b). Significant shifts in functional capacity were only detected when assessed as shared across different species as revealed by TaxSEA. Next, we applied TaxSEA to the faecal metagenome of T2D patients (*n* = 170) and controls (*n* = 174) [28]. Here, DA analysis identified 40 species that were significantly different between T2D and controls (FDR < 0.1, 26/40 increased in T2D; Supplementary Table S3). Using the per-species fold changes as input, TaxSEA detects a significant depletion of producers of two metabolites (FDR < 0.05, median absolute fold change >2.5×)—indole-3 propionic acid (IPA; Fig. 4c), and phenylalanine. (Supplementary Table S4). IPA is a microbially produced tryptophan metabolite which directly modulates insulin secretion and is being investigated as biomarker for T2D [33, 34]. Finally, we sought to evaluate whether the choice of DA analysis package to generate the input ranks for TaxSEA would impact detection of these signatures. After repeating each of the above DA analyses with ALDEx2 and fastANCOM, we found that the resulting TaxSEA *P*-values for each set were highly correlated (Pearson Cor > 0.97; Fig. 4d and e) and that the number and identity of the taxon sets significantly altered were identical across the three DA packages. Thus, TaxSEA can detect biologically relevant alterations in gut metagenomic data and is robust to the choice of DA analysis method.

We next evaluated existing taxon set enrichment analysis tools, MSEA and the TSEA module in the MicrobiomeAnalyst pipeline [14], to compare their performance with TaxSEA. As these tools differ from TaxSEA in both statistical methodology and database composition, we assessed them in their default implementations, reflecting how they are typically used in practice. For each of the IBD and T2D DA comparisons above, we extracted the list of DA species (FDR <0.1), split by fold change into ‘increased’ and ‘decreased’ species and then used each output as input for MSEA and TSEA. Neither MSEA nor TSEA detected any significantly (FDR < 0.1) enriched or depleted taxon sets. As noted above, the depletion of butyrate producers we detected was in part driven by taxa, which did not meet a threshold for statistical significance alone, highlighting the limitation of requiring a strict cut-off as part of an ORA test. Nonetheless, an ORA-based approach may be more suitable when taxa are analysed without associated ranks. Ngyuen and colleagues introduced CBEA [18] that generates enrichment scores for taxon sets in individual samples. While this approach provides valuable sample-specific insights, it requires taxon sets to be defined prior to analysis, which may limit its exploratory potential. However, combining CBEA with methods like TaxSEA, MSEA, or TSEA could enhance microbiome analysis by capturing both individual-level variation and broader group-level enrichment patterns.

While taxon set enrichment provides a powerful framework for detecting patterns in microbiome data, an inherent limitation is its dependence on factors such as database quality, taxonomic resolution, and detection thresholds. In this respect, a limitation of TaxSEA is its reliance on the quality and comprehensiveness of the underlying databases. Most microbiome-oriented databases are disproportionately focused on the human gut, which may limit applicability of TaxSEA to other microbial ecosystems. To address these limitations, we designed TaxSEA to be extensible, allowing users to add custom taxon sets or create their own database for testing. We also plan to expand its database coverage as new curated resources become available and we strongly encourage others developing microbiome databases to make them accessible via APIs to facilitate integration into enrichment analysis workflows whether that be via TaxSEA or other tools such as CBEA, MSEA, or MicrobiomeAnalyst.

An important consideration is that the threshold for detecting taxa in a dataset will influence enrichment results. Low-abundance features are inherently noisier and more susceptible to technical variability. Striking a balance between sensitivity and robustness is crucial, as overly permissive thresholds may introduce noise, while overly stringent filtering may exclude biologically meaningful taxa. We recommend leveraging the thresholds implemented in commonly used DA analysis packages, as they are optimized for their respective statistical frameworks. However, users of TaxSEA should carefully consider the trade-off between sensitivity and specificity, particularly when investigating rare or low-abundance taxa, where detection thresholds may have a greater impact on results.

Taxonomic resolution is also an important consideration when applying TSEA-like tools. While enrichment analysis can be performed at higher taxonomic ranks (e.g. order, family, or genus), the biological relevance of these groupings is highly variable. Some taxa exhibit functional consistency within a genus, while others show substantial species-level diversity, making enrichment results less informative at higher taxonomic levels.

A broader challenge in microbiome enrichment analysis is the lack of standardized benchmarking datasets. While we have provided a simple approach here using signal implantation, this may not be fully representative of true biological signals. To encourage wide adoption of TSEA-like methods, future research should prioritize the development of standardized datasets to enable rigorous comparison of tools. Furthermore, enrichment techniques should aim to better incorporate evolutionary relationships captured in phylogenetic trees. Implementing this approach at scale will require careful consideration of tree construction strategies and thresholds for defining phylogenetic groups.

## Conclusion

Here, we present TaxSEA, an R package that enables researchers to rapidly interpret and contextualize DA analysis results. While our focus has primarily been on the gut microbiota, TaxSEA is versatile and can be applied to any microbial community, including non-human-associated environments. This work demonstrates that TaxSEA successfully extracts implanted signals and recovers known associations from DA outputs. We encourage microbiome researchers to incorporate multiple taxon set analysis approaches, viewing ORA, KS-based enrichment, and sample-specific methods like CBEA as complementary tools that together offer a more comprehensive perspective on microbial community changes.

**Key Points**

- TaxSEA is a new R package designed for taxon set enrichment analysis, enabling microbiota researchers to explore patterns in differential abundance data.
- The package allows user to test for changes in custom sets, but also provides an in-built database with sets mined from five public microbiome databases (BugSigDB, MiMeDB, GutMGene, mBodyMap, GMRepoV2), covering disease signatures, metabolite producers, and previously reported associations.
- TaxSEA provides robust performance across DA pipelines and offers flexibility by allowing users to incorporate custom taxon sets for diverse microbiome datasets.

## Supplementary Material

Supplementary_Tables_bbaf173

## Data Availability

TaxSEA is open source and available as an R package on GitHub alongside all code and data for generating the results presented here (https://github.com/feargalr/TaxSEA). To support diverse research applications, we provide extensive documentation on the TaxSEA GitHub detailing how to utilize the TaxSEA-DB with other enrichment tools and utilize TaxSEA with sets defined based upon their taxonomic lineage. Benchmarking, code for creating figures and simulations can also be found on GitHub (https://github.com/feargalr/TaxSEA_benchmarking). An R shiny app can be found on GitHub (https://github.com/timrankin/Shiny-TaxSEA) and is also available online (shiny.taxsea.app). A python implantation of the core TaxSEA algorithm is also available via PyPi (https://pypi.org/project/TaxSEA-in-python/0.1.5/).
